# The Effect of Inflammatory Cytokines in Alcoholic Liver Disease

**DOI:** 10.1155/2013/495156

**Published:** 2013-12-09

**Authors:** Hideto Kawaratani, Tatsuhiro Tsujimoto, Akitoshi Douhara, Hiroaki Takaya, Kei Moriya, Tadashi Namisaki, Ryuichi Noguchi, Hitoshi Yoshiji, Masao Fujimoto, Hiroshi Fukui

**Affiliations:** Third Department of Internal Medicine, Nara Medical University, 840 Shijo-cho, Kashihara, Nara 634-8522, Japan

## Abstract

Alcohol is the most common cause of liver disease in the world. Chronic alcohol consumption leads to hepatocellular injury and liver inflammation. Inflammatory cytokines, such as TNF-*α* and IFN-*γ*, induce liver injury in the rat model of alcoholic liver disease (ALD). Hepatoprotective cytokines, such as IL-6, and anti-inflammatory cytokines, such as IL-10, are also associated with ALD. IL-6 improves ALD via activation of the signal transducer and activator of transcription 3 (STAT3) and the subsequent induction of a variety of hepatoprotective genes in hepatocytes. IL-10 inhibits alcoholic liver inflammation via activation of STAT3 in Kupffer cells and the subsequent inhibition of liver inflammation. Alcohol consumption promotes liver inflammation by increasing translocation of gut-derived endotoxins to the portal circulation and activating Kupffer cells through the LPS/Toll-like receptor (TLR) 4 pathways. Oxidative stress and microflora products are also associated with ALD. Interactions between pro- and anti-inflammatory cytokines and other cytokines and chemokines are likely to play important roles in the development of ALD. The present study aims to conduct a systemic review of ALD from the aspect of inflammation.

## 1. Introduction

**Figure 1:**
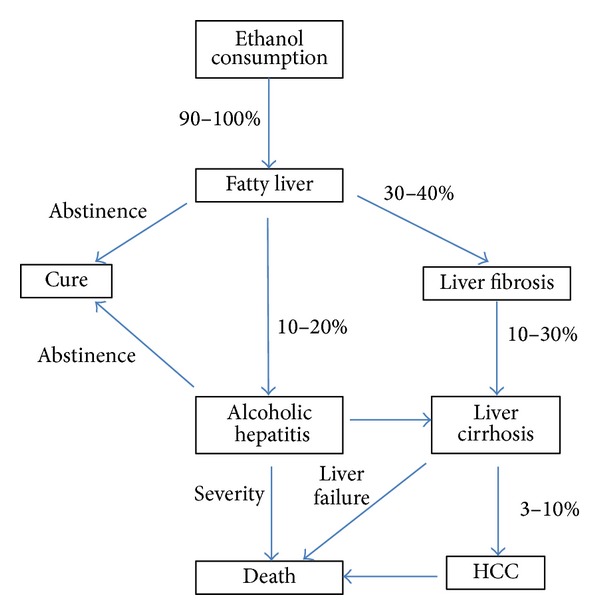
The natural history of alcoholic liver disease. Chronic ethanol consumption leads to fatty liver for more than 90%. But only up to 40% of this population develops more severe forms of alcoholic liver disease (ALD), including fibrosis and alcoholic hepatitis. Continuous ethanol consumption finally leads to liver cirrhosis or hepatocellular carcinoma and leads to death.

**Figure 2:**
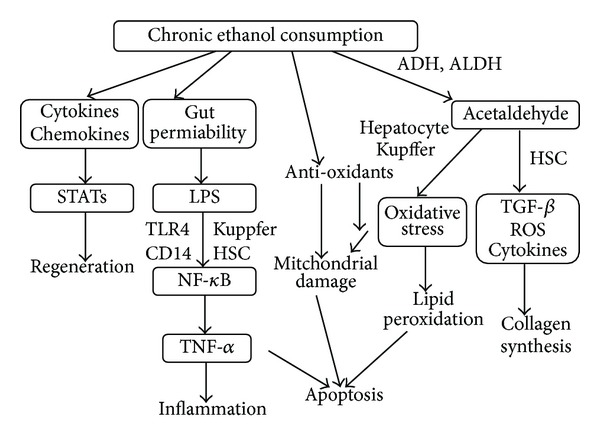
The pathogenic mechanisms of alcoholic liver disease. Chronic ethanol consumption promotes the translocation of LPS from the intestine to the portal vein, where it binds to the lipopolysaccharide-binding protein (LBP). STATs induces liver regeneration. Ethanol consumption alters the intracellular balance of antioxidants with subsequent decrease in the release of mitochondrial damage, leading to hepatic apoptosis. Hepatocytes and activated Kupffer cells are suggested to be the sources of oxidative stress, which are responsible for lipid peroxidation and further apoptotic damage. Activation of hepatic stellate cells also contributes to the production of TGF-*β*, ROS, and cytokines, leading to liver fibrosis.

Alcohol-related liver disease is a major cause of morbidity and mortality worldwide. Chronic alcohol consumption leads to hepatocellular injury, fat accumulation, and liver inflammation and sometimes leads to liver cirrhosis or hepatocellular carcinoma (Figure 1). The pathogenesis of alcoholic liver disease (ALD) is a consequence of chronic alcohol consumption. The clinical syndrome of ALD carries a particularly poor prognosis, such as liver cirrhosis [1] or hepatocellular carcinoma [2]. The pathogenesis of ALD is uncertain, but the relevant factors include metabolism of alcohol to toxic products, oxidative stress, acetaldehyde adducts, abnormal methionine metabolism, malnutrition, the activation of endotoxin, and impaired hepatic regeneration (Figure 2) [3]. Kupffer cells, the resident macrophages in the liver, play the role of an innate immune system; they produce various cytokines and are known to be involved in the pathogenesis of liver diseases [4]. The inflammatory cytokine, tumor necrosis factor-alfa (TNF-*α*), is involved in acute alcoholic liver injury [5]. Moreover, it is also well known that chronic alcohol consumption increases TNF-*α* production and leads to liver injury [6]. The consumption of alcohol leads to an augmented permeability of the intestinal membrane, which increases the portal concentration of blood endotoxin (lipopolysaccharide; LPS) [7]. This causes the Kupffer cells to be activated and exhibit enhanced sensitivity to LPS-stimulated inflammatory cytokine production [8]. Chronic alcohol consumption leads to injury of the hepatocytes by TNF-*α*, with consequent apoptosis and phagocytosis by the Kupffer cells. The Kupffer cells are activated by phagocytosing the apoptotic cells and their inflammatory cytokine production is increased [9]. Alcohol consumption promotes liver inflammation by increasing the translocation of gut-derived endotoxins to the portal circulation and activating Kupffer cells through the LPS/Toll-like receptor (TLR) 4 pathways. ALD is associated with imbalanced immune responses and increased production of proinflammatory cytokines and chemokines [10, 11]. Various cytokines are associated with ALD, including hepatoprotective cytokines, such as lnterleukin-6 (IL-6), and anti-inflammatory cytokines, such as IL-10 [12]. These two cytokines are produced by ethanol-induced LPS-stimulated Kupffer cells and can attenuate alcohol-induced liver injury. Inflammasome and several chemokines also contribute to ALD. Thus, the present paper aims to conduct a systemic review of ALD from the aspect of inflammation.

## 2. Metabolism of Alcohol

**Figure 3:**
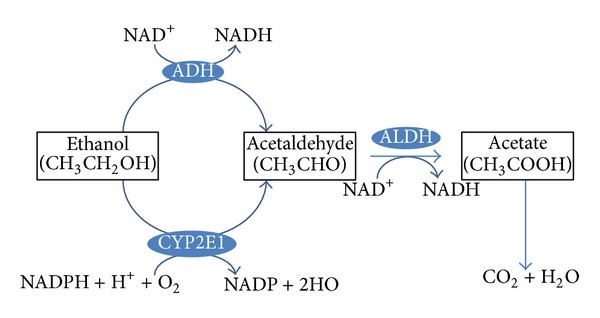
The pathway of ethanol metabolism. Ethanol is metabolized into acetaldehyde by alcohol dehydrogenase (ADH) and the microsomal enzyme cytochrome P450 2E1 (CYP2E1). The ADH enzyme reaction is the main ethanol metabolic pathway involving an intermediate carrier of electrons, namely, nicotinamide adenine dinucleotide (NAD^+^). Acetaldehyde is rapidly metabolized by aldehyde dehydrogenase (ALDH) in the mitochondria to acetate and NADH. And acetate is eventually metabolized in the muscle to carbon dioxide and water.

When alcohol is consumed, it passes from the stomach and intestines into the blood, a process referred to as absorption. Alcohol then enters the liver through the portal vein. We show the pathway of alcohol metabolism in Figure 3. In the liver, alcohol dehydrogenase (ADH), the key enzyme in alcohol metabolism, mediates the conversion of alcohol to acetaldehyde [13]. Acetaldehyde is rapidly converted to acetate by acetaldehyde dehydrogenase (ALDH) and is eventually metabolized in the muscle to carbon dioxide and water. There is another pathway independent of ADH, which is called the microsomal ethanol oxidizing system (MEOS) [14]. Alcohol is metabolized in the liver by the enzyme cytochrome P450 2E1 (CYP2E1). CYP2E1 is mainly expressed in the liver, with hepatocytes showing the highest expression, but it is also located in other organs, such as the brain and intestine. CYP2E1 is mainly located within the endoplasmic reticulum (ER) although it is also expressed in the mitochondria [15] and is increased after chronic alcohol consumption [16] and an increase in acetaldehyde. Acetaldehyde has a stronger toxicity than ethanol and leads to liver injury. Most of the alcohol consumed is metabolized in the liver, but the small quantity that remains is not metabolized and is excreted in the breath and urine.

### 2.1. TNF-*α*

TNF-*α* is a cytokine involved in systemic inflammation and is a member of a cytokine family that stimulates acute inflammation. TNF-*α* is produced by various types of cells in the body. In the liver, TNF-*α* is mainly produced by Kupffer cells, and TNF-*α* is also an important mediator in various physiological processes, such as inflammation, cell proliferation, and apoptosis [10]. The role of TNF-*α* as a critical inflammatory cytokine in the progression of ALD is now well known [5]. However, the mechanism of alcohol enhancement of TNF-*α* has not been clarified yet. Kupffer cells secrete inflammatory cytokines [4] and reactive oxygen species (ROS) [17], which activate cells such as hepatocytes, hepatic stellate cells, and endothelial cells [18]. In alcoholic hepatitis (AH), inflammatory cytokines, such as TNF-*α* or IL-6, induce liver injury [19]. After chronic alcohol consumption, Kupffer cells exhibit enhanced sensitivity to LPS-stimulated TNF-*α* production [20]. Elevated serum levels of TNF-*α* inducible cytokines or chemokines, including IL-6, IL-8, and IL-18, have also been reported in patients with AH [21]. Serum TNF-*α* is increased in patients with ALD and correlates with mortality. Administration of excessive ethanol to TNF-*α* knockout mice does not cause liver injury. Thus TNF-*α* is thought to be the main cytokine of inflammation. Furthermore, increased serum levels of TNF-*α* have also been noticed in rat models of nonalcoholic steatohepatitis (NASH) [22] and in patients with NASH [23]. TNF-*α* is associated with the development of liver injury in both ALD and NASH.

Recently, it has become known that platelet aggregation activity is associated with ALD. The platelet adhesive protein, von Willebrand factor (VWF), and its cleavage protease, ADAMTS13, have been gaining attention. In previous studies, our group showed that plasma ADAMTS13 activity decreased in ALD or severe AH and was inversely proportional to TNF-*α* [24–26]. Treatment with pentoxifylline, an inhibitor of TNF-*α* synthesis, improved the survival of patients with severe AH [27]. Anti-TNF-*α* antibodies prevented inflammation and necrosis in the rat model of alcohol feeding [6]. Anti-TNF-*α* antibody, infliximab, is also effective in severe AH patients [28]. Multiple cytokine modulator, Y-40138, is known to inhibit the production of inflammatory cytokines, such as TNF-*α* or IL-6, and to enhance the production of anti-inflammatory cytokines, such as IL-10. Our results showed that Y-40138 reduced the inflammatory cytokines in ALD [29]. These results suggest that TNF-*α* plays an important role in the progression of ALD.

### 2.2. IL-6

The role of IL-6 in ALD is complex and not well understood. It appears to have some beneficial effects on the liver. IL-6 may protect against hepatocyte apoptosis and participate in mitochondrial DNA repair after alcoholic liver injury [30, 31]. IL-6 may promote human Th17 differentiation and IL-17 production, therefore contributing to ethanol-induced liver inflammation. IL-6 is also released along with IL-10, TNF-*α*, and other cytokines by Kupffer cells after alcohol consumption. IL-6 and IL-10 are two cytokines that play roles in reducing alcoholic liver injury and inflammation through activation of the signal transducer and activator of transcription (STAT3) [12]. Elevated IL-6 is found in chronic alcohol-fed animals and in alcoholics, with or without liver disease [32]. On the other hand, IL-6 knockout mice fed chronic alcohol showed increased liver fat accumulation, lipid peroxidation, mitochondrial DNA damage, and sensitization of hepatocytes to TNF-*α* induced apoptosis, which was prevented by the administration of recombinant IL-6 [31, 33, 34]. Furthermore, blocking of IL-6 signalling in mice reduced the infiltration of neutrophils and mononuclear cells and inflammation [35]. These findings suggest that IL-6 has a protective effect at the early phase of ALD.

### 2.3. IL-10

IL-10 is an anti-inflammatory cytokine that controls the endogenous production of TNF-*α* during endotoxemia and reduces LPS stimulation when added exogenously [36]. IL-10 is produced by macrophages, lymphocytes, and Kupffer cells, and the liver is considered to be the main source of IL-10 production [37]. IL-10 decreases the production of proinflammatory cytokines, such as TNF-*α*, IL-1*β*, and IL-6, from activated macrophages or monocytes. IL-10 also possesses a hepatic protective effect on proliferation and fibrosis [38]. In the liver, Kupffer cells are the main producers of IL-10. Kupffer cells produce IL-10 in response to LPS stimulation and downregulate the release of TNF-*α* and IL-6. Endotoxin administration is an extensively studied model of IL-10 induction from monocytes and macrophages [39]. Human monocytes activated by LPS are able to produce a high level of IL-10 in a dose-dependent manner [40]. The activated monocytes inhibit production of proinflammatory cytokines, such as TNF-*α*, IL-6 and IL-1*β*. Moreover, proinflammatory cytokine levels were also increased by LPS treatment in IL-10 knockout mice [41]. Alcohol-fed IL-10 knockout mice have increased hepatic and systemic inflammatory conditions, and LPS enhanced alcohol-induced liver injury compared with wild-type mice [42]. On the other hand, IL-10 knockout mice have a reduced fatty liver and lower serum AST and ALT levels after ethanol feeding compared with wild-type mice [42]. This may be because IL-10 knockout mice have elevated levels of IL-6, and STAT3 activation in the liver, which lead to steatosis and hepatocellular damage. At the early stage of ALD, inflammation involving IL-6/STAT3 has a protective effect against alcoholic steatosis and liver injury. These findings suggest that IL-10 as well as IL-6 plays a protective role in the progression of ALD.

### 2.4. Other Interleukins

Nuclear regulatory factor kappa B (NF-*κ*B) is a protein complex that controls the transcription of DNA and a central regulator of cellular stress in all cell types in the liver. NF-*κ*B plays a key role in regulating the immune response to infection and in both acute and chronic inflammation. Activation of NF-*κ*B in rats can induce the expression of IL-1*β*, which increases the expression of proinflammatory molecules [43, 44]. IL-1*β* and IL-6 were found to be essential for the induction of Th17 lymphocyte differentiation from human naive CD4+ T cells [45]. Furthermore, LPS-stimulated human monocytes induced Th17 polarization of naive CD4^+^ T cells in an IL-1*β* signalling-dependent manner. IL-8 is a critical proinflammatory cytokine involved in many steps of neutrophil mobilization, from bone marrow to tissue infiltration or activation. IL-8 is induced by TNF-*α* and by ligands for TLRs via the activation of NF-*κ*B. Serum IL-8 is highly elevated in patients with AH and is linked to neutrophil infiltration. In contrast, IL-8 is only moderately elevated in alcoholic cirrhosis patients or alcoholics. IL-17 plays a key role in enhancing the host immune response against microorganisms as well as in autoimmune diseases [46]. IL-17 stimulates multiple types of nonparenchymal hepatic cells to produce proinflammatory cytokines and chemokines [47] with a relatively weak TNF-*α*-like function. Furthermore, IL-17 can act with other cytokines to activate NF-*κ*B and induce IL-8. Recently it was shown that patients with ALD had higher IL-17 plasma levels compared with healthy subjects [48]. The functions of Th17 cells are also mediated via the production of IL-22. IL-22 is a member of the IL-10 family of cytokines and plays an important role in promoting hepatocyte survival and proliferation [49]. IL-22 administration to alcohol-fed mice also prevented liver steatosis and liver injury through the activation of hepatic STAT3 [50].

IL-1*β* is also a potent proinflammatory cytokine [51]. In both animal model and patient with ALD, the levels of pro-IL-1*β* are significantly increased in the liver and in the serum [52, 53]. IL-1*β* is produced as inactive pro-IL-1*β* in response to inflammatory stimuli, including both microbial products and endogenous danger-associated molecules. IL-1*β* gene expression and synthesis of pro-IL-1*β* occur after activation of pattern recognition receptors (PRRs). IL-1*β* acts in an autocrine or paracrine manner via the type I IL-1 receptor (IL-1R1). Activation of IL-1R1 is inhibited by its binding to the IL-1 receptor antagonist (IL-1Ra). Treatment with IL-1Ra significantly improves symptoms in patients with rheumatoid arthritis [54], or autoinflammatory syndromes.

## 3. Toll-Like Receptors

**Figure 4:**
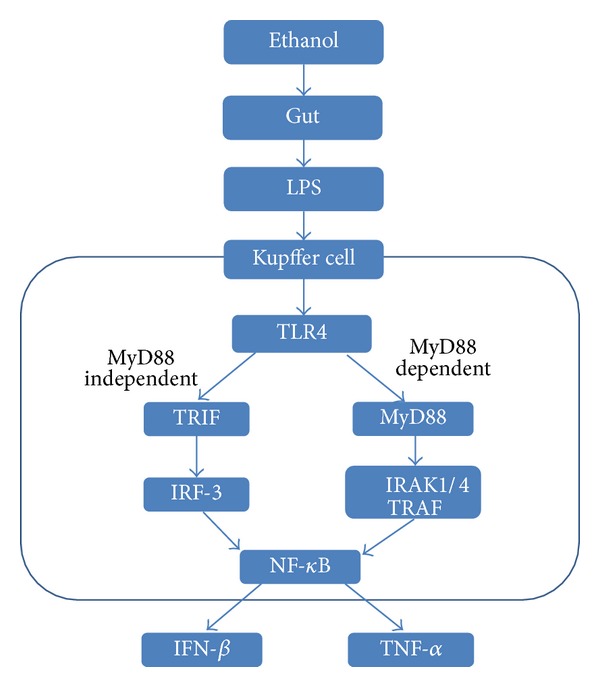
Toll-like receptor 4 signaling pathway in alcohol consumption. Ethanol promotes the translocation of lipopolysaccharide from the gastrointestinal lumen to the portal vein, where it binds to the lipopolysaccharide-binding protein. In Kupffer cells, lipopolysaccharide binds to CD14, which combines with TLR4 activating multiple cytokine genes. TLR4 are activated by MyD88 dependent or independent manner, leading to secretion of TNF-*α* or IFN-*β*.

Toll-like receptors (TLRs), a family of PRRs, are transmembrane proteins originally identified in mammals on the basis of their homology with Toll, a Drosophila receptor that contributes to the production of antimicrobial peptides that act against microorganism invasion in the fly. 11 TLRs have been identified in humans and 13 in the mouse [55]. TLRs recognize pathogen-derived molecules, such as structural components unique to bacteria, virus, and fungi, and activate inflammatory cytokines and type I interferon (IFN) production. TLRs are expressed on the surface of immune cells, such as macrophages, dendritic cells, and nonimmune cells, including epithelial cells. Expression of TLR1, 2, 6, 7, and 8 was elevated in chronic ethanol-feeding model. The treatment with ethanol resulted in sensitization to liver inflammation and damage by TLR1, 2, 4, 6, 7, 8, and 9 ligands due to increased expression of TNF-*α* [56]. However, deficiency in TLR2 had no protective effect in a chronic ethanol-feeding mouse model [57]. TLRs play important roles in the pathophysiology of a variety of liver diseases [58], which may attribute to wide expression of TLRs on hepatocytes [59]. TLR4 is a functional receptor expressed on the surface of macrophages and various other types of cells that transmit endotoxin signals. Cluster of differentiation 14 (CD14) is a protein that is a component of the innate immune system. CD14 binds to LPS, thereby subsequently presenting it to TLR4 and MD-2, which activate the intracellular signalling pathway via myeloid differentiation factor 88 (MyD 88), resulting in NF-*κ*B activation [60]. Both MyD88 and TRIF (MyD88 independent) signalling are associated with TLR3 and TLR4 (Figure 4). Recruitment of the TRIF adapter activates phosphorylation of interferon regulatory factor 3 (IRF3) that results in type I IFN production [61]. It is known that mice deficient in IRF3 or TLR4 expression are protected from alcohol-induced liver inflammation and hepatocyte injury [62]. The LPS/TLR4 signalling pathway consists of activation of transcription factors, such as NF-*κ*B, which induces proinflammatory cytokine expression in the Kupffer cell. In the liver, TLR4 is expressed not only on innate immune cells such as Kupffer cells, but also on hepatocytes, hepatic stellate cells, sinusoidal endothelial cells, and biliary epithelial cells. Blockade of TLR4 or CD14 reduces liver pathology and inflammation in a mouse model of alcoholic liver injury [63, 64], which indicates the importance of the TLR4 signalling pathway. LPS recognition by TLR4 expressed on hepatic stellate cells and sinusoidal epithelial cells also contributes to the progression of ALD [65]. Alcohol stimulates Kupffer cells and monocytes to produce increased TNF-*α* in response to endotoxin [66]. In previous studies, our group showed that endotoxemia plays an important role in the initiation and aggravation of ALD through the enhancement of proinflammatory cytokines, including IL-6, IL-8, and TNF-*α* [67, 68]. Hepatic expression of TLR1, 2, 4, 6, 7, 8, and 9 mRNA was increased in the mouse model of chronic alcohol feeding [69]. Alcohol feeding also resulted in sensitization to liver damage and inflammation because administration of TLR1, 2, 4, 6, 7, 8, and 9 ligands resulted in increased expression of TNF-*α* mRNA [69]. Acute alcohol exposure inhibited TLR4 signalling in macrophages after alcohol treatment in mice leading to decreased LPS-induced TNF-*α* production [70]. In ALD, TLR3 activation in HSCs and Kupffer cells plays an antagonistic role against the TLR4-mediated signal pathway via the production of IL-10 [71].

A certain double-stranded RNA virus, a ligand of TLR3, triggered the expression of IL-10 through IRF3 signaling [72] and that activation of TLR3 signaling induced IRF3 activation [73]. TLR3 and IL-10 participate in the suppression or killing of activated HSCs and Kupffer cells in a variety of models of liver injury. Recent investigations suggest that TRIF-regulated IRF3 binds to the promoter region of the TNF-*α* gene and upregulates transcription in chronic ethanol-exposed macrophages contributing to alcohol-induced steatosis [74]. These findings suggest that TLRs play a “gate keeper” role in ALD.

## 4. Chemokines

Members of the CXC family of chemokines include IL-8 and growth-regulated *α*-protein (Gro-*α*). These mediators attract polymorphonuclear leukocytes which are the predominant inflammatory cells that infiltrate the livers of patients with ALD. In patients with AH, expression of these chemokines in the liver correlates with the severity of portal hypertension and patient survival [75, 76].

CCL2, referred to as monocyte chemotactic peptide-1 (MCP-1), is a member of the CC chemokine family. Its expression can be induced in many cell types, including inflammatory cells, hepatocytes, and hepatic stellate cells. CCR2 is the only known receptor for CCL2 and is expressed on monocytes, T lymphocytes, and basophils [77]. MCP-1 regulates adhesion molecules and proinflammatory cytokines TNF-*α*, IL-1*β*, and IL-6 [78]. The pivotal role of MCP-1 in ALD was recognized by showing higher amounts of MCP-1 as compared to other CC chemokines, macrophage inflammatory protein 1*α* (MIP-1*α*), and MIP-1*β*, in the liver and mononuclear cells of patients with AH [79]. And MCP-1 is important in the modulation of proinflammatory cytokines [80]. Deficiency of MCP-1 protects mice against ALD, independent of CCR2, by inhibition of proinflamma-tory cytokines and induction of fatty acid oxidation, linking chemokines to hepatic lipid metabolism [81].

## 5. Inflammasomes

The inflammasome is a multiprotein oligomer consisting of caspase-1, PYCARD, and NALP that mediate the response to cellular danger signals activating and recruiting inflammatory cells. Procaspase-1 is activated by the inflammasome and cleaves pro-IL-1*β* into the bioactive IL-1*β* [82]. The inflammasome also promotes the cleavage of pro-IL-18 into IL-18 to induce IFN-*γ* secretion and natural killer cell activation, cleavage and inactivation of IL-33 [83, 84]. Inflammatory stimuli also drive activation of cytosolic caspase activation and recruitment domain (CARD) that recruit ASC and caspase-1 to assemble into the inflammasome. Inflammasome and IL-1*β* are activated in ALD patient or rodent animal model [85]. Recent studies demonstrated mRNA expression of several inflammasomes in the liver thus suggesting that inflammasome activation is a component of the liver pathophysiology in ALD [86].

## 6. Oxidative Stress

Oxidative stress is caused by excess ROS production, which leads to apoptosis and necrosis. ROS can also lead to a free radical chain reaction with unsaturated fatty acids generating toxic lipid intermediates. Oxidant stress is a pathogenic factor for the onset of ALD and nonalcoholic fatty liver disease (NAFLD). In vivo models of alcohol infusion induce lipid peroxidation because of increased free radical formation and decreased hepatic antioxidants, such as glutathione (GSH) [87]. In addition to GSH, other liver antioxidants, such as vitamin A, vitamin C, and bilirubin, and enzymes, such as superoxide dismutase and catalase, remove ROS. Moreover, treatment with an inhibitor of alcohol oxidation, such as 4-methylpyrazole, or an antioxidant, such as trolox, effectively prevented or reduced alcohol-induced toxicity, thereby demonstrating the importance of oxidant stress in the pathogenesis of ALD [88]. The catalytic activity of the cytochrome P450 enzymes requires oxygen activation, which results in the generation of ROS, such as the superoxide anion (O_2_^−^), hydrogen peroxide (H_2_O_2_), and the hydroxyl radical (•OH). Activated Kupffer cells are responsible for the release of various mediators, such as proinflammatory cytokines including TNF-*α*, IL-1, and ROS. ROS participates in inflammation and modulation of hepatocyte metabolism [89]. There is increased production of ROS in ALD. ROS can be released from a variety of sources, such as activated Kupffer cells, CYP2E1, and NADPH oxidase. NADPH oxidase can enhance the activation of NF-*κ*B and phosphorylate the ERK1/2 and p38 MAPK kinases that amplify Kupffer cell production of TNF-*α* [90]. Thus, ROS is highly involved in ALD.

## 7. Microflora Products

The importance of bacterial translocation in the pathogenesis of ALD has been demonstrated in many reports. Intestinal tight junction dysfunction [91–94] or bacterial proliferation [95, 96] caused by alcohol or its metabolites such as acetaldehyde enhance bacterial translocation into the liver, which induces the activation of Kupffer cells to release various proinflammatory cytokines and chemokines [97, 98] and increase acute LPS translocation. Chronic alcohol feeding causes structural changes in the gastrointestinal tract that might contribute to LPS translocation [99]. Continuous intragastric feeding of alcohol results in intestinal bacterial overgrowth and enteric dysbiosis, which is due to alcohol-induced downregulation of expression of several intestinal antimicrobial molecules [100]. In the intestine, disruption of tight junctions may lead to increased permeability to pathogens, which appears to be a common mechanism involved in the pathogenesis of ALD. Zonula occludens 1 (ZO-1), occludin, and claudin-5 are the transmembrane proteins that are expressed at the tight junction. Alcohol-treated mice showed loss of ZO-1, occludin, and claudin-5 expression in the colon. Gut-derived endotoxins play a crucial role in liver inflammation. Indeed, gastrointestinal permeability is higher in alcoholics than in normal [92, 101]. Alcohol-induced leaky gut results in the translocation of gram-negative bacteria from the intestinal lumen into the portal blood, elevating lipopolysaccharide (LPS) levels and triggering significant inflammation and liver injury [102–104]. The gut-liver axis is associated with alcohol-induced liver injury, both in experimental animal models and in patients of ALD. Intestinal sterilization with antibiotics prevents alcohol-induced liver injury [105]. Probiotic therapy reduces circulating endotoxin derived from intestinal gram-negative bacteria in ALD. These findings indicate that there are close interactions between the liver and intestinal bacteria in ALD.

## 8. Treatment

Abstinence from alcohol is the essential treatment for ALD [106, 107], but many ALD patients have difficulty remaining abstinent. Thus, there is an urgent need to develop novel therapeutic interventions. In the experimental setting, there are many animal models of ALD [108–111], but these have some advantages and disadvantages. In the clinical setting, alcoholics with acute hepatitis or cirrhosis have identifiable symptoms and receive treatment. Severe AH patients have a high mortality rate of about 50%, and those who survive have a 70% probability of developing liver cirrhosis. Nutritional supplementation is necessary for AH patients because of the prevalence of malnutrition. Only two pharmacological agents, which are corticosteroids and pentoxifylline, are recommended for treating AH. Both are aimed at reducing inflammatory conditions. Corticosteroids reduce cytokine production through transcriptional regulation. On the other hand, pentoxifylline achieves a similar effect through the inhibition of phosphodiesterase. These two agents have relatively strong side effects; hence, they are mainly used for cases of severe AH. New agents are showing promise for treating AH patients. Many antioxidants are effective in treating alcohol-fed animals. The consequent depletion of antioxidants leads to elevated oxidative stress that contributes to both the genesis and progression of ALD in animal models. However, the effectiveness of antioxidant therapy in human patients with ALD remains obscure. TLR3 activation might be a novel therapeutic strategy for the treatment of ALD. Probiotics are also effective in ALD. *Lactobacillus* reduces endotoxemia and improves liver injury in the rat model of ALD [112]. Moreover, some clinical studies have indicated the effectiveness of probiotics treatment. However, despite these improvements, an effective treatment for ALD has not yet been established. New treatment methods are required for ALD in the near future.

## 9. Conclusion

Alcohol is one of the most common causes of chronic liver disease in the world [113]. There are numerous factors, such as inflammation, oxidative stress, innate immunity, or fibrosis, that result in the development and progression of ALD. The inflammatory cytokines or chemokines appear to play an important role in ALD. The progression of alcohol-induced liver injury involves some immune cells and hepatocytes through the release of cytokines, chemokines, and inflammasomes. Kupffer cells play an important role in the early stage of ALD, producing TNF-*α* through TLR4. Based on the understanding of the pathogenesis of ALD, TNF-*α* is a key to developing new approaches to treatment, which has advanced very little since the introduction of corticosteroid therapy. The development of targeted therapies for ALD is hampered by poor knowledge of the molecular mechanisms involved in its development, particularly in humans, and by the perception that it is an addictive and a self-inflicting disease. We consider that more studies are needed to increase the understanding of the pathogenesis of inflammatory cytokines in order to open new therapeutic avenues for ALD.
